# Detection limits should be a thing of the past in gamma-ray spectrometry in general as well as in neutron activation analysis

**DOI:** 10.1007/s10967-016-4843-0

**Published:** 2016-06-02

**Authors:** Menno Blaauw

**Affiliations:** Reactor Institute Delft, Delft University of Technology, Mekelweg 15, 2629 JB Delft, The Netherlands

**Keywords:** Detection limits, Gamma-ray spectrometry, Peak search

## Abstract

In gamma-ray spectrometry with high-resolution detectors, full-energy peaks are often to be detected by a peak-search algorithm, with a threshold for detection. Detection limits can be derived from this. Detection limits are often computed along with measured activities or concentrations. When an analyte is not detected, the detection limit remains as the only available information. This leads to inhomogeneous datasets that are difficult or impossible to process correctly without introducing artefacts or biases. Here, it is proposed to determine peak areas at predetermined energies. An unbiased result with its uncertainty always results, obviating the “detection limit” concept.

## Introduction

### Currie’s detection limit definitions as related to gamma-ray spectrometry and NAA

In 1968, Currie [1] published a classic paper on detection limits where he presented derivations of formula’s for a critical limit *L*_*C*_ (the net signal level above wich a signal can be considered to have been reliably detected), a detection limit *L*_*D*_ (the net signal level at which a signal can be expected to be detected), and a quantification limit *L*_*Q*_ (the net signal level at which the measurement precision will be satisfactory for quantitative determination), based on Poisson counting statistics. All three are applicable in gamma-ray spectrometry and (I)NAA.

In high-resolution gamma-ray spectrometry, a peak search algorithm is often used, the sensitivity of which can be set using a threshold parameter that commonly represents a significance level in terms of standard deviations, i.e. the inverse of the relative peak area uncertainty. For example, assuming a perfect peak-search algorithm and a smooth continuum at the peak location, a threshold of “3” tends to imply that a peak with an expected net area of 0 will have a 0.13 % “false hit” probability of exceeding the threshold (using the cumulative normal distribution), and a peak that has an expected area with a relative uncertainty of 33 % is just at the threshold level, implying a 50 % probability of exceeding the threshold criterion. The precise relation between continuum level, peak area, peak width and detection probability depends on the peak-search algorithm used, as discussed in an earlier paper [2].

This threshold level corresponds to Currie’s *L*_*C*_. Often, it will be asked to determine “if an element is present” in a sample, and too often, the answer “no” is given if the peak-search algorithm has not detected a peak. To be correct, the answer should be “yes” in all cases—the measurement only serves to determine how much, as K. Heydorn used to teach the INAA community.

In order to “reliably” detect a signal, the reliability level needs to be stated. For example, if the signal is to be detected with a probability of 99.9 %, i.e. a “miss” probability of 0.13 %, the net signal must exceed the *L*_*C*_ level by at least 3 standard deviations (using the cumulative normal distribution, again depending slightly on the peak-search algorithm in practice). This, then, is the *L*_*D*_ level. In the examples given, the net *L*_*D*_ signal is 6 standard deviations above noise (3 for *L*_*C*_ and 3 additional ones for *L*_*D*_), so the relative 1 s.d. uncertainty in a peak area measured at the *L*_*D*_ signal level in the example would amount to 16 %.

**Fig. 1:**
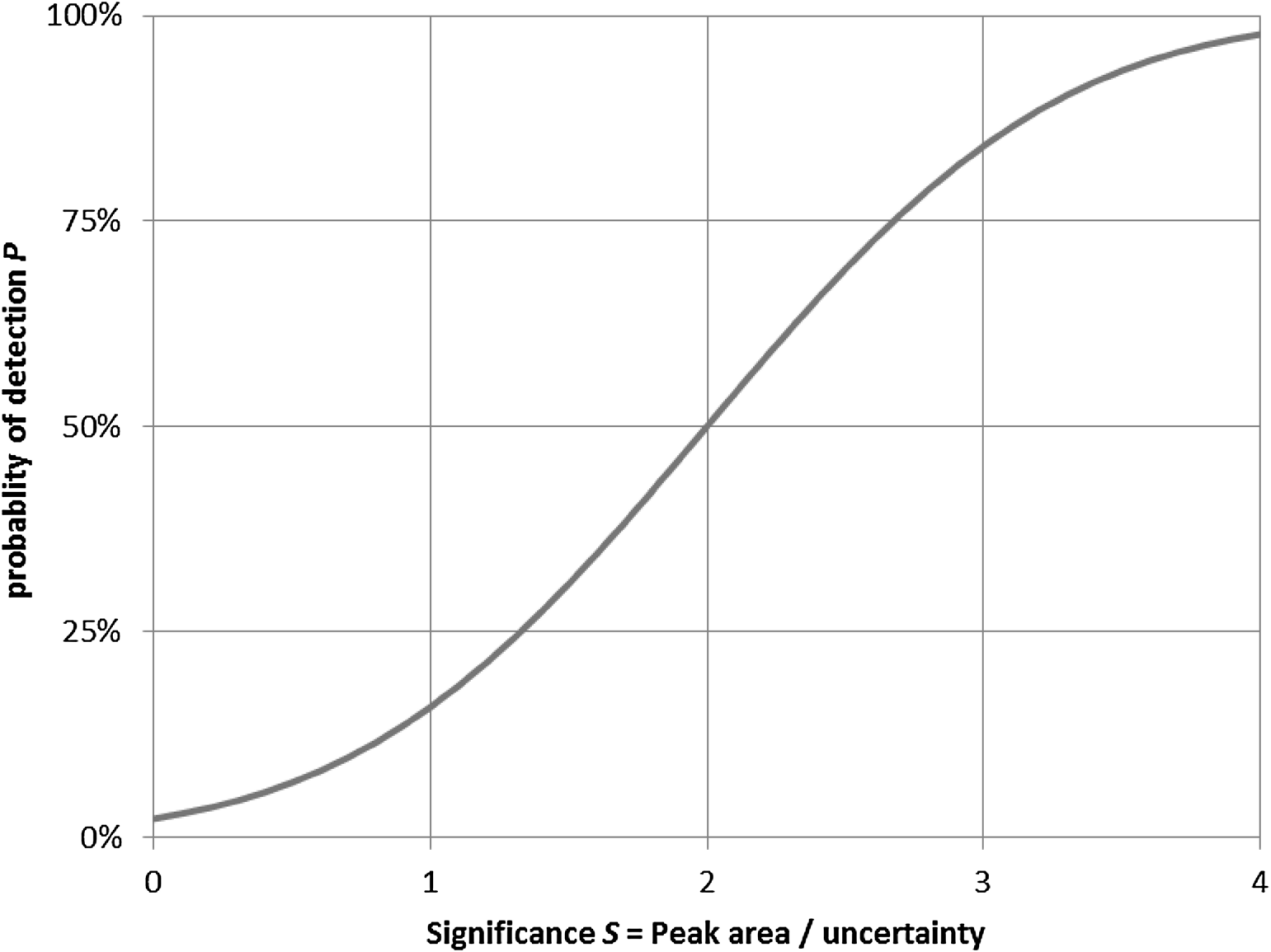
Probability of detection with the peak-search threshold set at 2. The “false hit” probability *P*(0) is 2.4 %, *L*_*C*_ corresponds to *S* = 2 where *P*(S) is 50 %, and the “miss” probability at S = 4, i.e. 1 − *P*(4), is 2.3 %. If this is the maximum “miss” probability required, S = 4 corresponds to *L*_*D*_

With the threshold set at “2” for a false hit probability of 2.3 % and the “miss” probability also to be set at 2.3 %, the net *L*_*D*_ signal would be 4 standard deviations above noise (2 for *L*_*C*_ and 2 additional ones for *L*_*D*_), so the relative 1 s.d. uncertainty in a peak area measured at the *L*_*D*_ signal level would then amount to 25 %. This example is illustrated in Fig. 1.

Too often, when element concentrations are wanted but not detected, the *L*_*C*_ or *L*_*D*_ are provided instead and regarded as an upper limit for the concentration present. This outcome might even be specified as *L*_*C*_/3 ± *L*_*C*_/3, because that concentration has a 99.9 % probability of yielding a signal strength below *L*_*C*_ and to go unnoticed as a consequence.

*L*_*Q*_, finally, is something to consider when concentrations are needed in the end with a certain minimum total uncertainty, when an analysis protocol is to be designed and/or the feasibility of the analysis in view of the desired total uncertainty is discussed. The mathematical relationship between expected peak area, peak width, continuum level and resulting relative peak area uncertainty are well know and can be used to this end.

### Disadvantages of the use of detection limits

All the above is straightforward and useful. However, it dates back to a time when many researchers, especially in the life sciences, would process (I)NAA data without taking the uncertainties into account, e.g. in unweighted linear regression or in the computation of Pearson’s correlation coefficient. Often, with other types of measurements, uncertainties in individual datapoints are not available, necessitating these unweighted data analysis techniques. Such researchers will then only want to use INAA concentrations measured above *L*_*Q*_, in order to be able to treat them all as equally precise.

However, researchers should take the specified uncertainty in each data point into account, when available. And the (I)NAA community, being able to specify good uncertainties from the counting statistics up, should provide them.

When doing epidemiological or environmental studies, large numbers of elements are often determined in large numbers of samples. The dataset is then to be treated with e.g. factor analysis later on. If one element has not been detected in a specific sample, either the sample or the element must be dropped from the statistical treatment of the dataset. This is undesirable, so “*L*_*C*_/3” or some such value is often used as a substitute for the absent concentration values. This is a dangerous approach for two reasons: First, these values have inherent, very large uncertainties and should never be used in an unweighted data treatment procedure. Second, the *L*_*C*_ level is determined from peak width and continuum level exclusively, and the (Compton) continuum level in the spectrum is a result of the presence of other elements, not of the element of interest! Using “*L*_*C*_/3” therefore leads to artefact correlations—even when the associated uncertainty would be taken into account. As the associated uncertainty describes the analytical process and not the property value measured, in statistical treatment of such measurement results weighted treatment (with weights inversely proportional to the to the variances) should be avoided, or systematic influences originating in the conditions of measurement may occur.

### How to do without detection limits

The solution proposed is to obviate the detection limit concept completely. This can be done as follows: When a gamma-ray spectrum has been measured, it is known where to expect the peak or peaks of the element of interest and additionally, their shapes are known from calibration measurements, so a peak-search algorithm is not needed at all. A peak can *always* be fitted to the spectrum, with the area and the continuum parameters as the only degrees of freedom in the fit. This will *always* yield a peak area with a well-determined uncertainty due to counting statistics, that can be used to calculate a concentration (or radionuclide activity) with its total uncertainty, taking all other sources of uncertainty into account. This way, there is no decision and no threshold for decisions in the process, and as a consequence, neither an applicable *L*_*C*_, nor an *L*_*D*_.

For this approach to work properly, the fitting procedure must not restrict peak areas to be positive, so that in the hypothetical case where an element is truly absent, half of the fitted peak areas will turn out negative, and so will the concentrations in the end. This is as it should be, because the average of the repeated determinations should go to zero as the number of determinations goes to infinity. If the element is present at a very low level, that average will go to the true, unbiased concentration only if negative individual results are take into account just like the positive ones.

Also, the peak areas and the concentrations will need to be stated with absolute uncertainties rather than relative ones, since peak area 0 and resulting concentration 0 might occur in a measurement, and an infinite relative uncertainty is meaningless as compared to an absolute uncertainty that can be expressed in useful numbers.

Since this approach *always* yields a concentration for every element, the resulting datasets will not suffer from contamination with detection limits, and all datapoints and elements can be fully used. However, the data processing will have to take the uncertainties in the individual data into account and must be able to cope with negative values. This is no problem—even in something as complicated as target-transformation factor analysis, it has been known how to do this for decades [3].

## Experimental

### Software modification

The in-house software for gamma-ray spectrometry was modified to fit peak areas at predefined energies. Standard lists of energies were defined for the standard analysis protocols used: very short, short, medium and long-lived nuclides. For each element, only the most important peak energies were included. From previous work [4], it is known that such catalogued energy values tend to be imperfect, and the corresponding peak positions need to be allowed to vary if the peak statistics are good enough. At the same time, to allow the user to clearly see what’s happening and to ensure that a peak area is determined for all elements to be determined in the protocol used, the peak energies reported in the end must be identical to the predefined ones.

Depending on the protocol used, the spectrum analysis software chooses the appropriate set of predefined energies. Using the energy calibration associated with the current spectrum, it converts these energies to positions. It then performs an ordinary peak search with the value of the threshold parameter 2 and merges the list of detected peak positions with the list of predined positions. If a detected peak matches a predefined peak to within ½ of the peak width (defined as the standard deviations of the local Gaussian peak shape), the two are deemed identical.

All peaks are then fitted. For the singlets with good statistics (i.e. relative peak area uncertainty smaller than 5 %), the peak position and shape parameters are fitted to the observed channel contents along with the peak area.

Finally, the peak positions found are matched to the predefined ones, where the predefined energy prevales over the measure energy.

The resulting peak areas are then interpreted in terms of elemental concentrations as usual [5].

### Test procedure

To test the performance of the whole procedure, the spectra of 91 blanks (10 mm height high-density polyethylene capsules type “W” purchased from Posthumus plastics), analyzed with the protocol for long-lived nuclides, were processed as described. Irradiation times, measurement times and detectors used varied from measurement to measurement. Predefined peak energies were used of 889 and 1120 keV for Sc, 1115.5 keV for Zn and 320 keV for Cr. The observed results for these elements were investigated. To do this, various statistics were determined:

- the number of times *N*_*det*_ that the uncertainty in the final result was smaller than 25 % (corresponding to a 97.5 % probability of detection if a peak-search algorihm at threshold 2 would have been employed). Also the number of times *N*_*pos*_ that the concentration turned out positive;
- the weighted average *x*_*w*_ of the concentrations found, calculated with
  1 $$ x_{w} = \frac{{\sum\nolimits_{i = 1}^{N} {{\raise0.7ex\hbox{${x_{i} }$} \!\mathord{\left/ {\vphantom {{x_{i} } {s_{i}^{2} }}}\right.\kern-0pt} \!\lower0.7ex\hbox{${s_{i}^{2} }$}}} }}{{\sum\nolimits_{i = 1}^{N} {{\raise0.7ex\hbox{$1$} \!\mathord{\left/ {\vphantom {1 {s_{i}^{2} }}}\right.\kern-0pt} \!\lower0.7ex\hbox{${s_{i}^{2} }$}}} }} $$
  where *N* is the number of determinations, *x*_*i*_ and *s*_*i*_ respectively are the measured concentration and its reported 1 s.d. uncertainty, the latter being dominated by counting statistics. Such weighted averages were also computed taking only the positive results into account, and taking only the results with uncertainties better than 25 % into account;
- the internal standard error of the mean, *S*_*int*_ for each dataset (all-inclusive, positive-only, better than 25 %), computed with
  2 $$ S_{\text{int}} = \sqrt {\frac{1}{{\sum\nolimits_{i = 1}^{N} {\frac{1}{{s_{i}^{2} }}} }}} $$
  where *m* is the unweighted average of the *x*_*i*_;
- the reduced *χ*_*r*_^2^, computed with
  3 $$ \chi_{r}^{2} = \frac{{\sum\nolimits_{i = 1}^{N} {z_{i}^{2} } }}{N} \quad using\,z_{i} = \frac{{x_{i} - x_{w} }}{{s_{i} }}. $$

## Results

The statistics on the results are shown in Table 1.

**Table 1:** Statistics on the results. amounts (*x*_*w*_, *S*_*int*_ and *S*_*ext*_) are specified in ng for Cr an Zn, in pg for Sc

	# results	*x*_*w*_	*S*_*int*_	*χ*_*r*_^2^
Cr (all data)	91	642.3	1.1	1.5
Cr (positive amounts only)	91	642.3	1.1	1.5
Cr (unc <25 % only)	91	642.3	1.1	1.5
Zn (all data)	91	65.2	0.7	4.6
Zn (positive amounts only)	89	65.2	0.7	4.6
Zn (unc <25 % only)	56	65.8	0.7	6.2
Sc (all data)	91	1.9	0.6	0.6
Sc (positive amounts only)	60	3.1	0.7	0.7
Sc (unc <25 % only)	0	–	–	–

## Discussion

### Chromium results

The results for chromium were obtained with uncertainties better than 25 % in all cases. The reported concentrations with their uncertainties are in reasonably good agreement with the final result *x*_*w*_, as indicated by the *χ*_*r*_^2^. The *χ*_*r*_^2^ does deviate from unity significantly at 91 degrees of freedom (*χ*_*r*_^2^-distribution at α = 0.001) and indicates the presence of unexplained variation, corresponding to a total uncertainty in *x*_*w*_ of 1.35 ng instead of 1.1 ng. If the implied additional uncertainty of 0.8 ng in *x*_*w*_ is considered to stem from chromium variability between capsules (or any source of variation other than counting statistics) the relative standard deviation of the *x*_*i*_ due to that variability is 1.1 %.

### Zinc results

About two-thirds of the zinc results had uncertainties better than 25 %. Only 2 out of the 91 turned out negative. Taking these into account does not affect the statistics of the whole dataset. Taking the results with uncertainties larger than 25 % into account has a small, insignificant effect on *x*_*w*_ and lowers *χ*_*r*_^2^ a bit. *χ*_*r*_^2^ deviates from unity very significantly in all cases, and, for the whole dataset, indicates the presence of an additional source of variation of 1.35 ng in *x*_*w*_, for a total of 1.5 ng. The relative standard deviation in the *x*_*i*_, corresponding to this unexplained variability, amounts to 20 %. Because the chromium results yielded an upper limit of 1.1 % for unexplained experimental sources of uncertainty, the 20 % for zinc must reflect between-capsule variation in zinc content.

### Sc results

All of the results for scandium had uncertainties worse than 25 %, and one-third turned out negative. Not taking the negative results into account has an effect on the statistics of the dataset, as expected. For example, the unbiased mean of 1.9 ± 0.6 pg indicates that the scandium concentration is above 0 with a 99.9 % reliability. The biased mean of 3.1 ± 0.7 pg makes that 99.9995 %. However, the difference between 1.9 ± 0.6 and 3.1 ± 0.7 is not significant at α = 0.05 since the zeta-score is only 1.3, and must exceed 2 to be significant at that confidence level. The *χ*_*r*_^2^ again deviates from unity significantly (at α = 0.005), and suggests slight overestimation of counting statistics uncertainties. A *χ*_*r*_^2^ of unity would have resulted if all reported uncertainties had been smaller by a factor of 0.8, leading to a best estimate for *x*_*w*_ of 1.9 ± 0.5 pg. No correlation is observed between the measured concentrations and their unceryainties.

### All results

Considering the results for chromium, zinc and scandium together, it is clear that the thresholdless approach yields useful data for all, including an element like scandium, that would not have been detected even once if a peak-search algorithm had been allowed to decide on its presence. A borderline element concentration like that of zinc, that would not have been detected in one-third of the cases, shows that the inclusion of those data does not affect the main statistics too much.

If these analyses had been performed the thresholdless way on samples in e.g. an epidemiological study, the resulting dataset would have been complete and useful for all three elements. In the traditional way, with the peak-search algorithm threshold, only two-thirds of the samples would have been complete for Cr and Zn and therefore useful, and Sc would have been missing entirely.

### Activity measurements

All the above can be applied to activity measurements of specific radionuclides, just like in (I)NAA. A list of predefined energies is needed, relevant to the radionuclide of interest, so that a peak-search algorithm need not be used to detect peaks at those energies for them to be fitted.

### Upper limits and quantification limits

In cases where an upper limit for a concentration (or activity) in a single analysis is desired, that upper limit can be computed from the measured value *x* and its uncertainty *s* at any confidence level. For example, *x* ± *s* corresponds to an upper limit of x + 2 s at α = 0.023, or x + 3 s at α = 0.001. It is also possible, using the normal distribution, to compute the probability that the concentration of an element (or the activity of a radionuclide) in the sample is above or below some e.g. legal limit.

In both cases, the availability of *x* ± *s* is more informative and therefore to be preferred over having only a detection limit at one’s disposal. It will be necessary to convey how important that uncertainty *s* is, in interpreting the data. It will also be necessary to explain why measured concentrations (or activities) can turn out negative, when it is know that real concentrations (and activities) can never be negative.

In order to communicate the performance characteristics of the procedure, the answer to the question “Can your technique or analysis protocol determine A at level x in the presence of B at level y?”, should never be “No”. Neither need the answer be “Yes, our quantification limit will then be *L*_*Q*_.”: the answer could always be “Yes, and with an uncertainty of z.” Uncertainties for various analyte levels can be provided. That information will be sufficient to assess if the technique will be good enough for the purpose at hand.

## Conclusions

The proposed method of thresholdless analysis of gamma-ray spectra has advantages as compared to traditional peak-search based procedures. More useful information is obtained, resulting in complete, unbiased datasets.

Since the proposed method is thresholdless, there is no critical decision level and no detection limit associated with it.

The limit of quantification remains applicable as a performance characteristic of the method. Alternatively, for any analyte in any matrix, the expected uncertainty in the measurement can be specified for any or a series of expected analyte levels.

The method is applicable to (I)NAA as well as to gamma-ray spectrometry in general.
